# Volatile Organic Compounds in Human Exhaled Breath to Diagnose Gastrointestinal Cancer: A Meta-Analysis

**DOI:** 10.3389/fonc.2021.606915

**Published:** 2021-02-26

**Authors:** Lijuan Xiang, Sihan Wu, Qingling Hua, Chuyang Bao, Hu Liu

**Affiliations:** ^1^ Department of Tumor Biotherapy (5th Ward of the Department of Oncology), Anhui Provincial Cancer Hospital, West District of The First Affiliated Hospital of USTC, Division of Life Sciences and Medicine, University of Science and Technology of China, Hefei, China; ^2^ Department of Oncology, The First Affiliated Hospital of Anhui Medical University, Hefei, China; ^3^ Department of Oncology, Affiliated Provincial Hospital of Anhui Medical University, Hefei, China; ^4^ Department of Oncology, Yijishan Hospital, Wannan Medical College, Wuhu, China

**Keywords:** volatile organic compounds, exhaled breath, gastrointestinal cancer, early diagnosis, meta-analysis

## Abstract

**Introduction:**

Human exhaled volatile organic compounds (VOCs) are being extensively studied for the purposes of noninvasive cancer diagnoses. This article was primarily to assess the feasibility of utilizing exhaled VOCs analysis for gastrointestinal cancer (GIC) diagnosis.

**Methods:**

PRISMA-based system searches were conducted for related studies of exhaled VOCs in GIC diagnosis based on predetermined criteria. Relevant articles on colorectal cancer and gastroesophageal cancer were summarized, and meta analysis was performed on articles providing sensitivity and specificity data.

**Results:**

From 2,227 articles, 14 were found to meet inclusion criteria, six of which were on colorectal cancer (CRC) and eight on Gastroesophageal cancer(GEC). Five articles could provide specific data of sensitivity and specificity in GEC, which were used for meta-analysis. The pooled sensitivity, specificity, diagnostic odds ratio (DOR), and area under the curve (AUC) were calculated based on the combination of these data, and were 85.0% [95% confidence interval (CI): 79.0%–90.0%], 89.0% (95%CI: 86.0%–91.0%), 41.30 (21.56–79.10), and 0.93, respectively.

**Conclusion:**

VOCs can distinguish gastrointestinal cancers from other gastrointestinal diseases, opening up a new avenue for the diagnosis and identification of gastrointestinal cancers, and the analysis of VOCs in exhaled breath has potential clinical application in screening. VOCs are promising tumor biomarkers for GIC diagnosis. Furthermore, limitations like the heterogeneity of diagnostic VOCs between studies should be minded.

## Introduction

Gastrointestinal cancer is of the leading causes of cancer deaths, approximately accounting for 22.2% of worldwide cancer related deaths (1). Till now, histological biopsy under endoscopy is still the predominant diagnostic method for gastrointestinal cancer. Since the early symptoms of gastrointestinal cancer are not specific, endoscopy yields unsatisfactory diagnostic rates, which also have shortages of being costly, painful and unsuitable for gastrointestinal cancer screening. Convenient, non-invasive and low-cost diagnostic methods are urgently needed for early cancer diagnoses and screening. Fecal occult blood testing, serum biomarkers and gastrointestinal barium angiography are commonly used in gastrointestinal cancer diagnosis, and fecal occult blood test is the most widely used and evaluable tests for current colorectal cancer screening. However, its clinical value is limited because of high false positive and negative rates. Serum biomarkers for gastrointestinal cancer, such as carcinoembryonic antigen (CEA) and Cancer antigen 19-9 (CA199) cannot play the expected diagnostic roles due to their poor accuracies. Gastrointestinal barium angiography can understand the overall location and size of the lesion, and the anatomic relationship with the entire organ, but there is a certain amount of radioactivity, and the procedures are troublesome. Therefore, noninvasive biomarkers are especially needed to be found for the purpose of diagnoses of gastrointestinal cancer.

Under normal physiological conditions, the concentrations of exhaled VOCs produced by the body’s metabolism were approximately 10^-12^ mol/L to 10^-9^ mol/L (2). In pathological conditions, metabolic abnormalities occurred and the production of VOCs increased significantly (3). Therefore, abnormal metabolic and pathological changes *in vivo* can be deduced by detecting increased VOCs. In the past decades, there have been extensive clinical studies to explore the relationship between the chemical compositions of the patients’ exhaled breath and clinical status of the patients. It is encouraging that exhaled VOCs have been used to diagnose some clinical conditions (4–6). As a new type of non-invasive examination method, it has shown broad application prospects in diagnosing pulmonary diseases (7–11), infections (12–16) and cancers (17–21), etc. It is generally believed that the production mechanism of VOCs is related to the excessive oxidative reactions taking places in cancer cells (22, 23), then spreading through the blood to the lung and respiratory tract.

Exhaled VOCs as a diagnostic tool for gastrointestinal cancer are of growing interest to scientists. However, most of the current researches are in the early stages lacking unified conclusions. Here, we systematically summarized the current knowledge on their potential clinical usages in early detection of gastrointestinal cancer and conducted a meta-analysis to evaluate their diagnosis power, hoping to build a stepping stone for future researches.

## Methods

### Search Strategies

This systematic review was completed in accordance with the Preferred Reporting Items for Systematic Reviews and Meta-Analysis (PRISMA) statement. PRISMA-based system searches were conducted (24) until 23 December 2018 in PubMed, EBSCO, ELSEVIER ScienceDirect, Wiley Online Library, and The Cochrane Library. At the same time, the references were followed for related reviews to obtain relevant information undiscovered. The terms “cancer OR tumor OR neoplasm OR malignant OR carcinoma”, “exhaled” and “VOCs” or “volatile organic compounds” were considered as keywords searching for related articles.

### Inclusion and Exclusion Criteria

The inclusion criteria of related studies about exhaled VOCs in GIC diagnosis were as below: (i)pathologically confirmed gastrointestinal cancer; (ii) trials that analyzed endogenous VOCs within exhaled breath to diagnose or assess cancer; (iii) clinical studies.

The exclusion criteria were as below: (i) no specific experimental details were provided; (ii) commentary articles rather than research articles. (iii) VOCs were analyzed not in exhaled breath but in breath condensate or other biofluid, including urine, serum, feces, and gastric content. In addition, articles that presented sensitivity and specificity data were included as criteria for meta-analysis.

### Data Extraction and Quality Assessment

Two reviewers independently screened and extracted data based on inclusion and exclusion criteria, discussed and resolved in case of disagreement. General Information such as authors, countries, participants, methodologies, techniques and experimental conclusions included in the study was extracted. Sensitivity and specificity that could be used for meta analysis was also extracted. The quality of included studies for meta-analysis was assessed using QUADAS-2 which was used for the quality assessment of diagnostic accuracy studies specially (25).

Methodological quality and risk of bias of included studies was determined by combining the Newcastle–Ottawa Scale (26) (NOS)。In this scale, each study was divided into three groups based on eight items: selection of study groups, comparability between groups, and determination of outcomes. The maximum score for each item was 1, but proportionality allowed for a score of 2. Total scores ranged from 0 to 9, with higher scores indicating better quality. The quality assessment was conducted independently by two authors (LJX and CYB) based on the Newcastle Ottawa Scale. The implementation of this assessment tool is discussed by both authors. The degree of agreement between the two authors was calculated by the other author (SHW). For the current study, we considered studies with a score of 7 or higher as high quality studies. Low-quality studies (Newcastle Ottawa score equal to or less than 4) were excluded.

### Statistical Analysis

META-DISC software(version 1.4) was adopted to evaluate the diagnostic values of articles included in meta-analysis. The source of heterogeneity was first evaluated, including threshold effects and non-threshold effects. The threshold effect was checked by the SROC curve plan: if it was in the “shoulder arm” shape, it indicated that there was a threshold effect; otherwise the opposite. Heterogeneity between studies was evaluated by applying the chi-square and I^2^ test, if P<0.05, I^2^>50%, which indicated the existence of statistical heterogeneity. As for the combination of effect quantities, when there was a threshold effect, the best method for data combination was to fit SROC curve and calculate AUC, or to apply other statistics such as Q index. If the heterogeneity was due to non-threshold effect, the analysis could be attempted in homogeneous subgroups. The publication bias of the included studies was assessed using the Deeks’ funnel plot of stata12.0. An asymmetric funnel plot was obtained when publication bias was present, i.e., a slope P <0.05.

## Results

### Description of Included Studies

**Figure 1:**
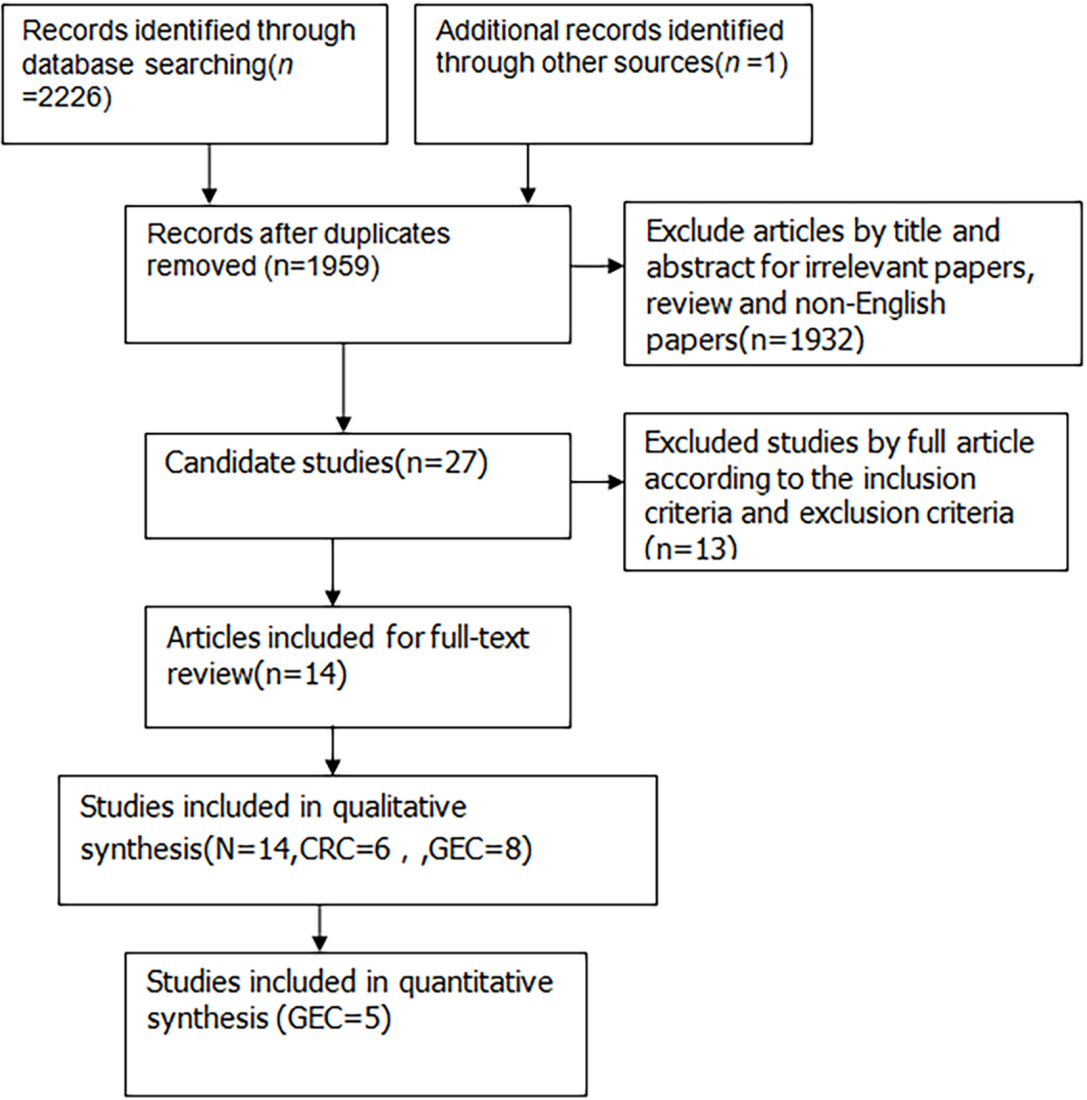
Flowchart of study selection for this meta analysis.

A total of 2227 articles were found using the search strategy described before among which 1,959 articles remained after removing duplicate articles. 1,932 studies were excluded from the title and abstract for using irrelevant papers, reviews and non-English papers. Thus, 27 studies were selected for full-text browsing. 13 studies were excluded for not meeting the inclusion and exclusion requirements. Thereby, 14 studies were included in the systematic description, including six in CRC and eight in GEC (shown in **Figure 1**). Five studies in GEC were able to conduct meta-analysis.

**Figure 2:**
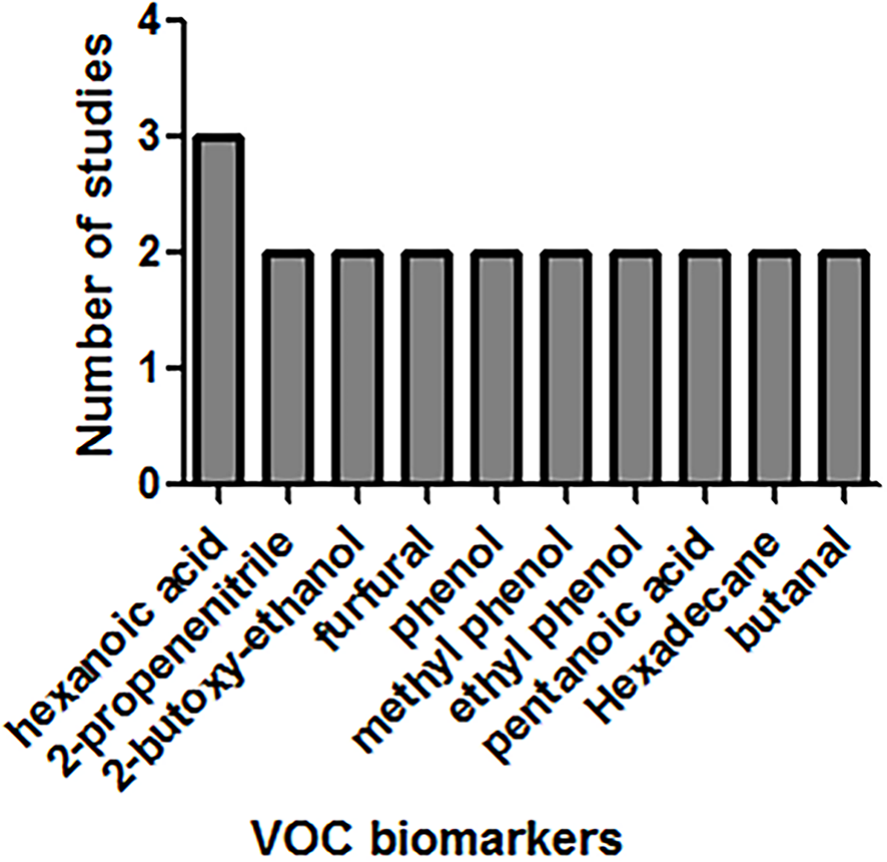
VOCs appeared in two or more GEC studies.

**Figure 3:**
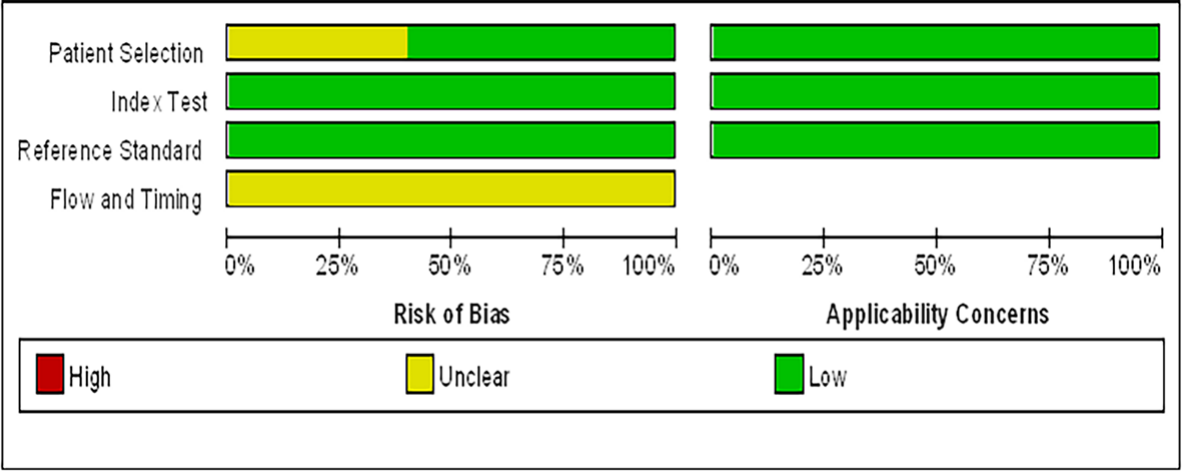
Graphical display for the Quality Assessment of Diagnostic Accuracy Studies-2 tool results: review authors’ judgements about each domain presented as percentages across included studies.

Data of the 14 studies on gastrointestinal cancer diagnoses by exhaled breath VOC were summarized comprehensively. Various techniques had been described to collect and analyze exhaled VOCs. Exhaled gas samples were usually collected temporarily in inert bags (including tedlar bags, mylar bags, nalophan bags and steel breath bags) or cans, volume varied between 20 ml and 4 L, and then analyzed directly; some of them used sorbent tubes to trap the exhaled gas before analysis because the gas could be stored in them for a long time without loss. The most commonly used detection technique was Gas Chromatography-Mass Spectrometer (GC-MS, n=8), usually combined with nano-sensor (n=5), and one combined with a probabilistic neural network (PNN). Four studies were analyzed using selected ion flow tube mass spectrometry (SIFT-MS), whilst one study used self-made proton transfer reaction mass spectrometry (PTR-MS). There were seven of the 14 reviewed studies used an leave-one-out cross-validation, but only two (27, 28) used an additional data set to verify the model. Basic information about the included studies of CRC and GEC was summarized in **Tables 1** and **2**, respectively. The potential biomarkers found in the exhalation of CRC and GEC patients are detailed in **Tables 3** and **4**. No consistent markers were found in the CRC study. It is worth noting that 10 VOCs appeared in two or more Gastroesophageal cancer studies, as shown in **Figure 2**, which partly reveals the repeatability of exhaled VOCs in the Gastroesophageal cancer field. Moreover, five studies capable of meta-analysis were evaluated with QUADAS-2 (**Figure 3**). The average Newcastle Ottawa score was 7.2 for all included studies (**Table 5**).

**Table 1:** Characteristics of included studies on exhaled VOCs for CRC diagnosis.

Author	Country	Objective	Participants	Breath collection	Volume	Analysis time	Technique	Targeted markers	Conclusion
Amal et al. (29)	Latvia	CRC screening	65 CRC, 22 AA or NAA, 122 HC	Tedlar bag +sorption tubes	750ml	Within 4 months	GC-MS+nanosensors	4 VOCs	Volatile marker testing by using sensor analysis is a promising noninvasive approach for CRC screening
Peng (30)	Israel	CRC diagnosis	30 LC,26 colon cancer,22 breast cancer, 18 prostate cancer, 22 HC	Mylar bags	750 ml	Within 4 days	GC-MS+nanosensors	6 VOCs	Exhaled VOCs are an inexpensiv, easy to use, portable, non-invasive methods of cancer diagnosis
Altomare (27)	Italy	CRC diagnosis	37 CRC and 41 HC; a further 25 subjects for validation	Tedlar bag	2 L	immediately	TD-GCMS+PNN	15 VOCs	Breath VOC analysis appears to have potential clinical application in colorectal cancer screening
Wang et al. (31)	China	CRC diagnosis	20 CRC and 20 HC	glass vials	20 ml	Within 3h	SPME-GC/MS	9 VOCS	Breath VOCs could represent an effective and convenient screening method for CRC diagnosis
Altomare (32)	Italy	CRC and polyps screening	15CRC, 15polyps,15HC	Tedlar bags	3L	immediately	PEN3 e-nose	Failure	It is impossible of the commercial e-nose as screening tool for patients with CRC and polyps.
Markaret al. (28)	London	CRC diagnosis and recurrence monitoring	50 CRC; 50 positive controls, 50 negative controls	Nalophan bags	2 L	1 h	SIFT-MS	propanal	This study suggests the association of a single breath biomarker with the primary presence and recurrence of CRC

NAA, non-advanced adenomas; AA, advanced adenomas; HRA, highrisk adenomas; LC, lung cancer; NA, not available; HC, healthy controls; SIFT-MS, selected-ion-flow-tube mass-spectrometry; SPME, solid-phase microextraction; GC-MS, Gas Chromatography-Mass Spectrometer; TD-GCMS, Thermal Desorption GCMS.

**Table 2:** Characteristics of included studies on exhaled VOCs for GEC diagnosis.

Author	Country	Objective	Participants	Breath collection	Volume	Analysistime	Technique	Targetedmarkers	Conclusion
Xu (19)	China	GC diagonosis	37 GC, 32 ulcers, 61 less severe conditions	Tedlar bag+ sorption tubes	4 L	4 months	GC-MS and nanosensors	5 VOCs	A nanomaterial-based breath test is a new and promising avenue to diagnose GC
Amal et al. (37)	Latvia	GC diagnosis(GC vsOLGIM)	99 GC, 325 the control group (OLGIM 0–IV)	Mylar bags	750 ml	Within 4 days	GC-MS and nanosensors	8 VOCs	Nanoarray analysis is a non-invasive screening tool for GC as well as for surveillance of the latter.
Zou (33)	China	Esophageal cancer diagnosis	29 esophageal cancer, 57 HC	Directly	At least 7s	Immediately	Home-made PTR-MS	7 kinds of ions	Exhaled VOCs may be a promising method in the esophageal cancer screening.
Kumar (34)	England	Esophageal and gastric adenocarcinoma diagnosis	48 esophageal, 33 GC and 129 noncancer controls	Nalophan bag	2 L	Within 1h	SIFT-MS	12 VOCs	Distinct exhaled breath VOC profiles can distinguish patients with esophageal and gastric adenocarcinoma from noncancer controls
Kumar (35)	England	EGC diagnosis	18 esophageal or gastric cancer, 18 positive control groups,17 HC	Nalophan bag	2 L	Immediately	SIFT-MS	4VOCs (EGC vs positive control), 4VOCs (EGC vs HC)	Results highlight the potential of exhaled VOCs as a noninvasive test to identify EGC.
Durán-Acevedo (36)	Colombian	GC diagnosis	14GC,15positive control and 1 undefined	Immediately or stored in adsorbent tubes	129 ml	Within 6 months	GC-MS and nanosensors	6 VOCs	Exhaled breath analysis is a new and nonintrusive methodology for early diagnosis of GC
Markar (37)	England	OGC diagnosis	163OGC,83 positive control and 89 HC	Steel breath bags	500 ml	Within 4 h	GC-MS and SIFT-MS	5 VOCs	The breath test demonstrated good diagnostic accuracy
Schuermans (38)	China	GC diagnosis	16 GC and 28 HC	Directly	3 min	Directly	Electronic nose	–	The e-nose has the capability of diagnosing GC based on exhaled air

PUD, peptic ulcer disease; EGC, esophago-gastric cancer; OLGIM, operative link for gastric intestinal metaplasia assessment; OGC, oesophagogastric cancer; HC, healthy controls; GC-MS, gas chromatography-mass spectrometer; SIFT-MS, selected-ion-flow-tube mass-spectrometry; PTR-MS, proton transfer reaction- mass spectrometry.

**Table 3:** Expiratory biomarkers between CRC patints and healthy patients.

Author	Biomarkers
Peng et al. (30)	10-(1-butenylidene)bisbenzene; 1,3-dmethy benzene;1-iodononane;[(1,1-dimethyiethyl)thio]acetic acid; 4-(4-propylcyclohexyl)-40 cyano[1,10-biphenyl]-4-yl ester benzoic acid; 2-amino-5isopropyl-8-methyl-1-azulenecarbonitrile
Altomare (27)	Nonanal;4-Methy1-2-pentanone; Decanal;2-Methylbutane;1.2-Pentadiene, 2-Metyipentane,3-Methylpentane;Methylcyclopentane;Cyclohexane; Methylcyclohexane;1,3-Dimethylbenzene; 4 Methyloctane; 1,4-Dimethylbenzene;A(4-methylundecane, RT=11-3);B(timethyldecane, RT=13-2)
Wang et al. (31)	Cyclohexanone, 2,2-dimethyldecane; dodecane; 4-ethyl-1-octyn-3-ol;ethylailine; cydoctyimethanol; trans-2-dodecen-1-ol;3-hydroxy-2,4,4-timethylpentyl2-methyipropanoate; 6-t-buty4-2,29,9-tetramethyl-3,5-decadien-7-yne
Amal et al. (29)	Acetone,6 ethyl acate, ethanol, 4-methyl octane
Markar (28)	Propanal

Variables A and B are compounds that are curentynot wlldentfied.

**Table 4:** Expiratory biomarkers between GEC patients andnon-cancer patients.

Author	Biomarker
Xu (19)	2-propenenitrile,2-butoxy-ethanol, furfural,6-methyf1-5-hepten-2-one, isoprene
Kumar (35)	Hexanoic acid, phenol, methyl phenol, ethyl phenol
Kumar (34)	Pentanoic acid, hexanoic acid, phenol, methyl phenol, ethyl phenol, butanal, pentanal, hexanal, heptanal, octanal, nonanal, decanal
Amal (21)	2-Propenenitrile; Furfural;2-Butoxy-ethanol; Hexadecane; 4-Methyl Octane; 1,2,3-Tri-methyl-benzene;α-methyl-styrene;2-Butanone
Durán-Acevedo (36)	Trans-2,2-dimethyl-3-decene; Octadecane, M-xylene;Hexadecane; 1-Cyclohexyl-2-(cyclohexylmethyl)pentane; Eicosane
Markar (37)	Butyric acid, pentanoic acid, hexanoic acid, butanal, decanal

**Table 5:** Newcastle–Ottawa Scale scores.

study	Selection				Comparability	Outcome/Exposure			score
	Is the case definition adequate	Represent-ativeness of the cases	Selection of Controls	Definition of Controls	Comparability of cohorts on the basis of the design or analysis	Ascertainment of exposure	Same method of ascertainment for cases and controls	Non-Response rate	
Durán-Acevedo (34)	○	○		○	○	○	○	○	7
Amal (21)	○	○		○	○○	○	○	○	8
Kumar (32)	○	○		○	○○	○	○	○	8
Schuermans (36)	○	○			○	○	○	○	6
Xu (19)	○	○		○	○	○	○	○	7

### Exhaled VOCs for CRC

The first reported attempt to identify CRC with exhaled VOCs was Peng et al. (30), the results showed that six VOCs could be used to distinguish colon cancer from healthy controls (HC). They also studied the relationship of exhaled VOCs between lung, colon, breast, prostate cancers and HC by GC-MS and nanosensor. Both techniques could distinguish “healthy” and “cancerous” by breathing, and furthermore, nano-arrays could also be used in differentiation among different cancer types. Amal et al. (29), also used GC-MS and nanosensor techniques to screen CRC. Four VOCs (Acetone, ethylacetate, ethanol, and 4-methyl octane), identified by GC-MS, showed significant differences between CRC group and control group in both studies. Additionally, this study also included adenoma patients as an independent group for comparative analysis, which were also found that could be effectively distinguished from either the cancer group or the control group.

In a clinical study, Altomare et al. (27) used thermal-desorber (TD) GC-MS and a probabilistic neural network (PNN) to analyze exhaled VOCs in 37 people with CRC and 41 controls. After the exclusion of unusual compounds and removal of ineffective variables, 15 potentially high discriminate power compounds were left behind. The pattern of applying PNN with 15 compounds showed significant performance with an accuracy of over 75%. Similar promising results were shown by Wang et al. (31) using solid-phase microextraction (SPME)-GC/MS, which was used to discriminate between 20 CRC patients and 20 HC with high diagnostic performance through nine significant VOCs. Markar et al. (28) analyzed exhaled breath samples from 50 CRC, 50 positive controls, and 50 negative controls patients using SIFT-MS. Seven compounds were shown to be statistically different between the cancer group and the control group, of which only propanal (NO+) had a meaningful increase in the cancer group related to the control group. When using a threshold of 28 ppbv, the sensitivity and specificity of CRC diagnosis were found to be as high as 96% and 76%, respectively. Beyond that their research group also explored the VOC changes associated with CRC recurrence after surgical resection. After surgery, propanal reduced to the desired levels consistent with the control patients, and with CRC recurrence, its levels significantly increased. Altomare et al. (39) picked 48 patients which belonged to the CRC group of 52 patients already monitored in their previous study, and 55 HC also confirmed the potential application of VOCs pattern in CRC patients for clinical follow-up. Eleven compounds were selected for discriminating disease-free patients after curative surgery from CRC patients before surgery with a sensitivity of 100% and a specificity of 97.92%. Disease-free follow-up patients could also be well recognized from HC by the same VOCs pattern. This study further suggested the potential association of exhaled VOCs with cancer screening and secondary prevention.

At the same time, a reliability assay of commercial electronic nose (PEN3 e-nose) as a screening tool for CRC and polyp patients found that it was impossible to discriminate the tested groups by using supervised or unsupervised statistical methods (32). They analyzed that the sensor’s unspecific response to the presence of defined exhaled VOC may be the reason for random classification of subjects to each group.

### Exhaled VOCs for GEC

A few of studies found that an accurate Gastroesophageal cancer diagnosis was possible using profiles of VOCs (**Table 2**). Kumar et al. (35) applied SIFT-MS to quantify the exhaled VOCs in three groups of patients, GEC, benign disease of the esophagus or stomach, and healthy cohort, 17 VOCs had been investigated in this study. Four of 17 VOCs were found to be in statistically significant different between cancer and positive control groups. Comparison of VOC profiles between cancer and HC also revealed a similar differential pattern. ROC analysis was used for the combination of the above four VOCs to discriminate the Gastroesophageal cancer group from positive controls, with an integrated AUC of 0.91. Similar to the previous study, Kumar et al. (33) performed breath analysis on two groups of patients with esophageal (N=48) or gastric adenocarcinoma (N=33) group, noncancer control group including Barrett’s metaplasia (N=16), benign upper gastrointestinal diseases (N=62) and a normal upper gastrointestinal tract (N=51) by SIFT-MS analysis, twelve VOCs were revealed in these two groups with significant higher concentration differences. The results showed that differentiated exhaled VOCs could distinguish esophageal or gastric adenocarcinoma patients from noncancer patients satisfactorily.

Durán-Acevedo et al. (36) utilized GC-MS and nanosensors to analyze breath samples from 14 gastric cancer (GC) and 15 positive control. A significantly higher concentration of six VOCs was found in the cancer group compared to the control. And the nanosensors were able to discriminate gastric cancer patients from controls achieving a sensitivity of 100% and a specificity of 93%. Xu et al. (19) analyzed breath samples from 37 gastric cancer, 32 ulcers, and 61 less severe conditions, and found that five VOCs of gastric cancer and/or peptic ulcer patients were significantly increased compared with less severe gastric conditions. The nanomaterial-based sensors analysis results shown that it could well separate gastric cancer, gastric benign disease, gastric ulcer and less severe conditions. And the results were not affected by confounding factors. In addition, early stages GC (I and II) and late stages GC (III and IV) could also be distinguished (89% sensitivity; 94% specificity). A similar study was conducted by Amal et al. (37, 40), and 968 breath samples from 484 patients (including 99 with GC) were analyzed by GC-MS and nanosensors, respectively. It was found that cancer patients and high risk patients had distinctive respiratory markers. GC-MS revealed eight of 130 different VOCs differed in various groups. The combination of cross-reactive nanoarrays and pattern recognition methods found that the gastric cancer group and the control group (OLGIM 0-IV) could be distinguished with a sensitivity of 73% and a specificity of 98%. And the subgroups also could be distinguished effectively.

Markar et al. used the previous published data sets to create a 5-VOCs diagnostic model with a diagnostic accuracy of 90%. In this study, they utilized GC-MS and SIFT-MS to verify the feasibility of OGC patients and controls detection by measuring VOCs in the exhaled breath. The result showed certain volatile components of exhalation had potential for non-invasive OGC with a diagnostic accuracy of 0.85 (35). Schuermans (38) analyzed the exhaled VOC profiles from 16 GC and 28 HC with electronic nose (It is manufactured by eNose in Zutphen, The Netherlands. It contains three micro hot plate metal oxide sensors and a pump). The results showed the e-nose were able to discriminate patients from controls achieving a sensitivity of 81% and a specificity of 71%, with an accuracy of 75%.

Zou et al. (33) utilized home-made PTR-MS to compare breath samples from 29 esophageal cancer patients and 57 healthy people. It had been found that seven kinds of ions in the breath mass spectrum could better distinguish between the two groups of patients with a sensitivity of 86.2% and a specificity of 89.5%, respectively. Five of the seven ions reduced and the rest two increased when esophageal cancer patients compared with the healthy people. The AUC of ROC analysis was 0.943.

### Data Analysis of Meta-Analysis

Five of the eight studies on the diagnostic Gastroesophageal cancer provided gastric cancer diagnostic study data, which met the quantitative analysis criteria. Sensitivity and specificity were extracted from the five studies (**Table 6**). The methodology of analysis technique used had no effect on the results because they only used different means to analyze the same class of substances to diagnose the same disease.

**Table 6:** Studies included in the meta-analysis.

Author	Technique	Sample size	Sensitivity	Specificity	Accuracy
Xu (19)	Nanosensors	37 GC vs 93 nonmalignant gastric conditions	89%	90%	90%
Amal (21)	Nanosensors	99 GC vs 325 OLGIM0-IV	81%	90%	88%
Kumar (34)	SIFT-MS	33 GC vs 113 noncancer control	88%	89%	83%
Durán-Acevedo (36)	Nanosensors	14 GC vs 15 noncancer control	100%	93%	97%
Schuermans (38)	Electronic nose	16 GC and 28 healthy controls	81%	71%	75%

**Figure 4:**
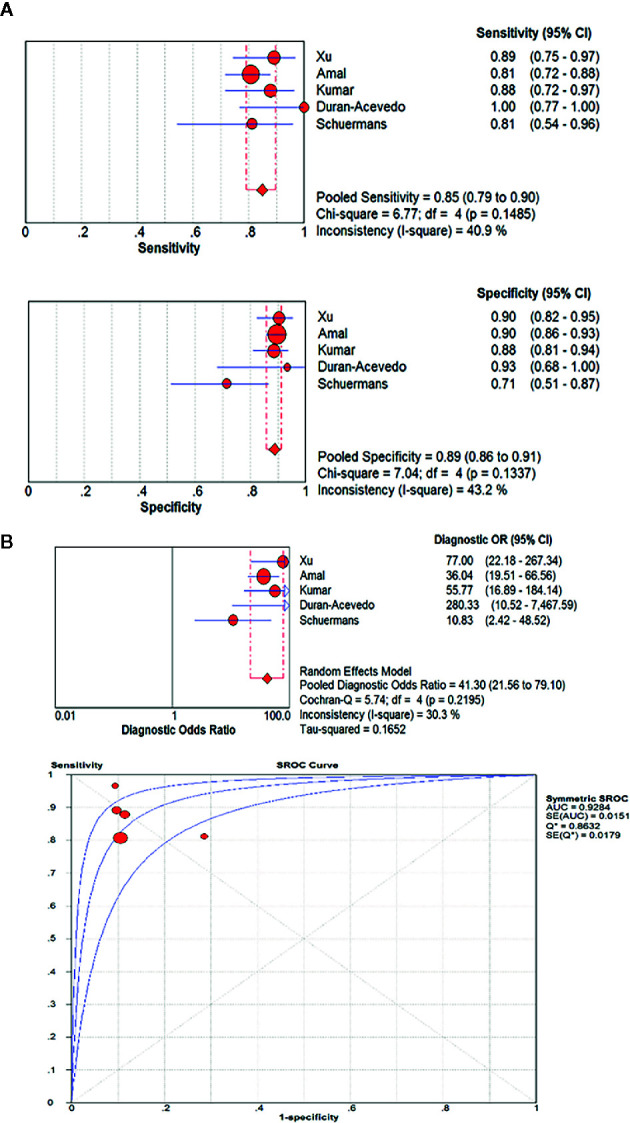
The pooled sensitivity, specificity, diagnostic odds ratio and SROC curve of exhaled VOC profiles in GC diagnosis. (OR, odds ratio; CI, confidence interval; DOR, diagnostic odds ratio.) **(A)** The pooled results reported a mean sensitivity of 85% (79% to 90% CI) and specificity of 89%(86% to 91% CI). **(B)** The mean (95% CI) pooled DOR was 41.30(21.56–79.10). The area under the SROC curve(AUC) was 0.93.

A pooled analysis of the included five studies showed no heterogeneity in sensitivity (chi-squared=6.77, p=0.1485; I^2^ of 40.9%) or specificity(chi-squared=7.04, p=0.1337; I^2^ of 43.2%). Therefore, the fixed effect model was applied. The pooled results reported a mean (95%CI) sensitivity of 85% (79% to 90% CI) and specificity of 89% (86% to 91% CI). The mean (95% CI) pooled positive likelihood ratio (PLR) was 6.65 (4.41–10.02), which indicated that GC patients are approximately six times more likely to have a GC-related exhaled VOC profiles than individuals without GC. And the mean (95% CI) pooled negative likelihood ratio (NLR) and diagnostic odds ratio (DOR) was 0.19 (0.14–0.26) and 41.30 (21.56–79.10), respectively. The area under the SROC curve (AUC) was 0.93. More information was available in **Figure 4**.

### Publication Bias and Heterogeneity

**Figure 5:**
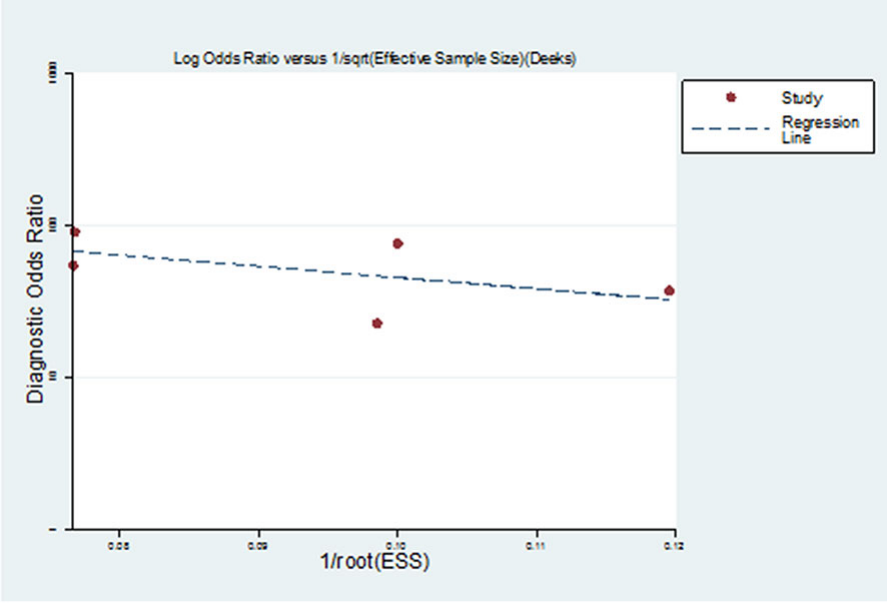
Deeks funnel plot of studies included in the meta-analysis. There was no published bias in Deeks funnel plot of studies included in the meta-analysis.

As a result of the Deeks funnel plot, there was no published bias (P=0.362>0.05) (**Figure 5**), although this result was limited by the small number of studies included in the meta-analysis. One of the main causes of heterogeneity in diagnostic studies was the threshold effect. To evaluate the diagnostic threshold, ROC curve plan and the Spearman’s correlation coefficient between sensitivity and 1-specificity was calculated. ROC curve plane scatter chart was not “shoulder arm” distribution and the correlation coefficient was 0.100, (p = 0.873), suggesting that there was no heterogeneity from the threshold effect.

## Discussion

Measurement of VOC signal profiles in exhaled gases is intended to identify a unique fingerprint/odor bolt associated with certain diseases potentially contributing to early diagnosis and improving survival rate. The principle behind exhaled VOCs in cancer detection is that cancer-associated VOCs in the tissue are released into the bloodstream and eventually exhaled by alveolar gas exchange (41). Researches mentioned in this review suggested that certain possible VOC biomarkers could be used to identify GIC with considerable sensitivity and specificity, which will make up for the deficiency of current GIC screening methods and shed light on the current development of GIC diagnosis. Additionally they are also expected to play a role in monitoring cancer recurrences. Our meta-analysis showed that VOCs were used to distinguish between GC and nonmalignant gastric conditions with sensitivity of 85%, and specificity of 89%. And GC patients were approximately six times more likely to have a GC-related exhaled VOC profiles than individuals without GC. DOR and AUC values were 41.30 and 0.93, respectively. These data indicate that exhaled VOC fingerprint analysis may be a promising approach for GC diagnosis. In addition, studies using VOCs to identify CRC and healthy patients displayed a diagnostic accuracy greater than 80% (27–29).

### The Possible Biochemical Origin

Next, we focus on the possible biochemical origin of the VOCs markers that has been identified in two or more studies of GEC. Among the 10 VOCs, we found that phenol and its derivatives were significantly elevated in exhaled breath of GEC patients, namely phenol, methyl phenol and ethyl phenol. Phenol is one of the decomposition products of tyrosine (42), so we speculate that the increased demand and overuse of amino acids in tumor tissue may be responsible for the increase of phenol in exhaled breath of GEC patients. Studies had shown that plasma tyrosine levels were significantly reduced in patients with GEC (43–45), to some extent indicate the credibility of the results. In addition, changes in the concentration of phenolic compounds were also observed in gastric cancer urine (46) and gastric contents (47). However, the corrupting effect of intestinal bacteria on protein products also produces phenols. The study of Ahmed WM et al. (48) further confirmed that the metabolism of commensal microbes and pathogenic bacteria are likely to affect the composition of exhaled VOCs. Therefore, it is necessary to analyze the potential biological sources of volatiles.

Xu et al. (19) and Amal et al. (40) both found that 2-acrylonitrile significantly increased in the exhaled breath of GEC patients, compared to the non-GEC group. Mochalski P et al. found that the levels of 2-propenenitrile was related to the occurrence of H. pylori through an ANOVA test on non-cancerous tissue samples (49). Pylori infection, as one of the important causes of gastric cancer, which providing clues to the production mechanism of this compound. As a class 2B carcinogen, 2-acrylonitrile can be produced from tobacco combustion or automobile exhaust. Despite studies showing that smoking, diet and other confounding factors do not affect the experimental results (28, 29, 37). But it cannot be ignored that the composition of exhaled breath was susceptible to indoor air pollutants. So confounding factors should be controlled as much as possible.

Studies showed that the production of exhaled VOCs was associated with lipid peroxidation (22, 23); alkanes are mainly produced by peroxidation of polyunsaturated fatty acids (PUFAs), which contain multiple conjugated double bonds and methylene-CH2- groups (50) provides the basic conditions for the production of alkanes. Therefore, it is possible to detect the increase in hexadecane relative to non-cancerous tissues in exhalation of GEC patients.

The above is just a tentative explanation of the increased VOCs in GEC exhaled breath. The specific mechanism needs to be further studied.

## Research Limitations

Although the above studies on the use of exhaled VOCs for the diagnosis of GIC have all achieved positive results, there is great heterogeneity among the diagnostic VOCs obtained. Next we will analyze the possible reasons.

### Influence of Detection Technique

The preference of instrument detection range may be one of the reasons for the large heterogeneity of the analyzed VOCs. The traditional method of VOC analysis is mainly GC-MS, which can give qualitative and quantitative information about exhaled VOCs (19, 35, 37). However, there are certain restrictions on the use of this technology, which is expensive and complicated. Inevitably, the use of VOCs for clinical diagnosis and monitoring requires more feasible technical support. Nanomaterial-based sensors, also called electronic noses, a new analysis method has been used for diseases diagnosis research due to their smaller size, easier to use, less expensive, as well as the advantages of sensitive, fast, and responsive. The commonly used electronic nose consists of a nonselective electrochemical sensor arrays and an appropriate pattern recognition software. This technology records distinct patterns of different VOCs present in a gas mixture in response to an unknown component, excluding the need to chemically separate or identify individual components. However, the possible detection limitations of the sensor system must take into account.

### Influence of Collection Method

It is worth noting that those studies reviewed are diverse in the use of procedures for collection and anatomical collection sites. A study showed that expiratory flow rate and breath holding time could affect the level of exhaled breath significantly (51). Unfortunately, most studies lack consistency in these parameters. Furthermore, common techniques for sample storage include the use of containers, such as inert bags, glass bottles, will also introduce contaminants and cause the loss of volatile organic compounds during storage (52, 53), although it turns out that Tedlar bag is superior to the rest of the polymers in terms of background emissions, especially stability and reusability. It is also important to note that the exhaled gas includes the alveolar gas exchanged with blood, and the respiratory dead space air, that which is, gas present on the airway or on the top of the alveoli that cannot exchange with blood. Should respiratory dead space air be removed during gas collection? Four research groups (29, 30, 37) filtered the dead space air and only collected the alveolar breath to analysis, but most research groups used mixed gases directly. Further research is needed to determine whether it is necessary to filter the dead space air.

### Influence of Endogenous and Exogenous Volatile Organic Compounds

Since exhaled breath includes a variety of endogenous and exogenous VOCs, we need to confirm that these VOCs are related to cellular metabolism itself, not to the microenvironment of indirect metabolic pathways in cancer or other *in vivo* (human or animal). A study showed that the composition of exhaled breath was susceptible to indoor air pollutants, and at the same time, as many as 86 substances were detected in exhaled breath, which were significantly associated with smoking habits (54). So confounding factors should be controlled as much as possible. Moreover, the metabolism of commensal microbes and pathogenic bacteria are likely to affect the composition of exhaled VOCs (48). So It is necessary to analyze the potential biological source of volatiles. Studies showed that H. pylori uses host cholesterol to defend against antibiotics (55), which leads to an increase in cholesterol biosynthesis, and isoprene as an intermediate in cholesterol biosynthetic pathways will increase accordingly, which may explain the observed higher levels of exhaled isoprene in patients with gastric ulcers (19). In addition to the above, more complicated situations need to be considered. Some gases are found to be exchanged in the airways or alveoli according to their blood solubility. Blood high solubility gases are exchanged in the airways, while low exchanges in the alveoli (56–59). Therefore, we should re-evaluate the diagnostic value of vocs with significant differences.

Additionally, the more commonly encountered shortcomings are the small sample size and the relatively single disease currently studied. there are certain limitations in the clinical complexities. Most research is still limited to the study of exhaled breath biomarkers, showing the potential of breath analysis in the field of gastrointestinal diagnosis. Lack of further large-sample clinical validation studies.

## Conclusion

Gastrointestinal cancer is one of the common malignant tumors with a high mortality rate. Therefore, early diagnosis and screening are the key to improving their prognosis. As a non-invasive tool, exhaled VOCs have shown great potential in gastrointestinal cancer diagnosis. which will make up for the shortcomings of current GIC screening methods and provide inspiration for the current development of GIC diagnosis. However, most of the volatiles detected by the current researches have large heterogeneity, so it is particularly important to establish a standard gas collection process and find a portable and accurate detection platform. At the same time, it is necessary to analyze the possible biochemical origin of these volatiles and clarify some endogenous and exogenous interference factors.

At present, the origin of the acquired diagnostic volatiles is mostly in the stage of analysis and inference, and the specific molecular metabolism mechanism is not clear, resulting in a lack of sufficient theoretical support. In addition, to use these volatiles as early tools for clinical diagnosis, large-scale multi-center clinical validation studies are still needed.

## Data Availability Statement

The original contributions presented in the study are included in the article/supplementary material. Further inquiries can be directed to the corresponding author.

## Author Contributions

LX and SW completed the design, data analysis and manuscript writing. HL contributed to the conception and revised the manuscript. QH and CB provided suggestions for revision of the manuscript. All authors contributed to the article and approved the submitted version.

## Funding

National Natural Science Foundation of China (Grant No. 81472750; 82061138004) and Natural Science Foundation of Anhui Province (Grant No. 1508085MH171).

## Conflict of Interest

The authors declare that the research was conducted in the absence of any commercial or financial relationships that could be construed as a potential conflict of interest.
